# Modeling functional connectivity changes during an auditory language task using line graph neural networks

**DOI:** 10.3389/fncom.2024.1471229

**Published:** 2024-11-15

**Authors:** Stein Acker, Jinqing Liang, Ninet Sinaii, Kristen Wingert, Atsuko Kurosu, Sunder Rajan, Sara Inati, William H. Theodore, Nadia Biassou

**Affiliations:** ^1^The Integrative Neuroscience of Communication Unit, National Institute on Deafness and Other Communication Disorders, National Institutes of Health, Bethesda, MD, United States; ^2^Biostatistics and Clinical Epidemiology Service, National Institutes of Health Clinical Center, National Institutes of Health, Bethesda, MD, United States; ^3^Department of Radiology and Imaging Sciences, National Institutes of Health Clinical Center, National Institutes of Health, Bethesda, MD, United States; ^4^Clinical Epilepsy Service, National Institute of Neurologic Disorders and Stroke, National Institutes of Health, Bethesda, MD, United States

**Keywords:** graph theory, graph neural network, line graph, functional connectivity, machine learning, functional MRI

## Abstract

Functional connectivity (FC) refers to the activation correlation between different brain regions. FC networks as typically represented as graphs with brain regions of interest (ROIs) as nodes and functional correlation as edges. Graph neural networks (GNNs) are machine learning architectures used to analyze FC graphs. However, traditional GNNs are limited in their ability to characterize FC edge attributes because they typically emphasize the importance of ROI node-based brain activation data. Line GNNs convert the edges of the original graph to nodes in the transformed graph, thereby emphasizing the FC between brain regions. We hypothesize that line GNNs will outperform traditional GNNs in FC applications. We investigated the performance of two common GNN architectures (GraphSAGE and GCN) trained on line and traditional graphs predicting task-associated FC changes across two datasets. The first dataset was from the Human Connectome Project (HCP) with 205 participants, the second was a dataset with 12 participants. The HCP dataset detailed FC changes in participants during a story-listening task, while the second dataset included the FC changes in a different auditory language task. Our findings from the HCP dataset indicated that line GNNs achieved lower mean squared error compared to traditional GNNs, with the line GraphSAGE model outperforming the traditional GraphSAGE by 18% (*p* < 0.0001). When applying the same models to the second dataset, both line GNNs also showed statistically significant improvements over their traditional counterparts with little to no overfitting. We believe this shows that line GNN models demonstrate promising utility in FC studies.

## 1 Introduction

The interconnectivity of the human brain is a critically important component in the understanding of the neural basis of behavior, in general, and in linguistic behavior in particular. Patients with strokes that disrupt the pathways between designated ROIs have been reported to have specific language deficits, such as Broca’s and Wernicke’s aphasia as well as conduction and global aphasia. However, understanding the mechanisms by which these language deficits arise has been elusive but may be essential in developing novel approaches in neurorehabilitation. Therefore, the development of neurocomputational models of the neural basis of language processing in the healthy human brain may be an important step in better understanding the mechanisms that result in specific language deficits following brain injury. With the advancements in biotechnology over the last 30 years, neuroscientists have been able to image the human brain in action. The advancements of computational power over the last decade have the tremendous potential to advance our understanding of language processing following brain injury through the use of computer simulations. For these reasons, the merger of machine learning algorithms and non-invasive neuroimaging techniques that analyze brain activity in real time is a crucial step in the convergence of cognitive and behavioral neurology and computational neuroscience towards the burgeoning modern field of clinical neuroscience. Often, neuroimaging studies divide the brain into regions of interest (ROIs). Functional connectivity (FC) studies look at the relationships between those different brain regions by calculating the correlations in activation between them (Bullmore and Sporns, 2009; He and Evans, 2010). Functional magnetic resonance imaging (fMRI) is a non-invasive neuroimaging method in which MRI images are combined with blood oxygen level-dependent (BOLD) signals, allowing us to map BOLD effects in different parts of the brain and thus measure brain activity on a per-ROI basis.

Previous fMRI-based static FC studies have shown that language related connection strengths between ROIs change during language tasks (Tran et al., 2018; Doucet et al., 2017). Certain neurological conditions, such as strokes, are known to both impact the patient’s FC and their language abilities (Tao and Rapp, 2020; Berthier, 2005; Boes et al., 2015). This makes the neurocognitive modeling of FC processes underlying language crucial to understanding and predicting functional neural responses under conditions of central nervous system (CNS) injury (Kamarajan et al., 2020; Nebli et al., 2020). One area of mathematics which is particularly important in modeling FC is graph theory, which has traditionally allowed us to view ROIs as nodes and functional correlations as edges (Bullmore and Sporns, 2009; He and Evans, 2010).

There are examples of machine learning models using resting state FC data in predicting medical or demographic information, such as identifying depression in people with Parkinson’s disease and identifying an infant’s age; or classifying static FC changes in neurodegenerative diseases (Lin et al., 2020; Kardan et al., 2022). Graph neural networks (GNNs) are a type of neural network architecture designed to incorporate graph structures as data (Scarselli et al., 2009). Given the inherent graph-based nature of the brain, GNNs – including those that incorporate less-traditional definitions of nodes as edges, as are found in hypergraphs – are an intuitive step in analyzing network neuroscience data (Nebli et al., 2020; Kardan et al., 2022; Zong et al., 2024; Zuo et al., 2024). We assessed the use of GNNs in predicting FC changes for a language task, specifically an auditorily presented language comprehension task as proof of concept that GNN can serve as a powerful computational tool in predicting language related static functional connectivity spatial neuronal changes. We specifically included the comparison of traditional GNNs and line GNNs to predict changes in auditory language comprehension based FC.

**Figure 1:**
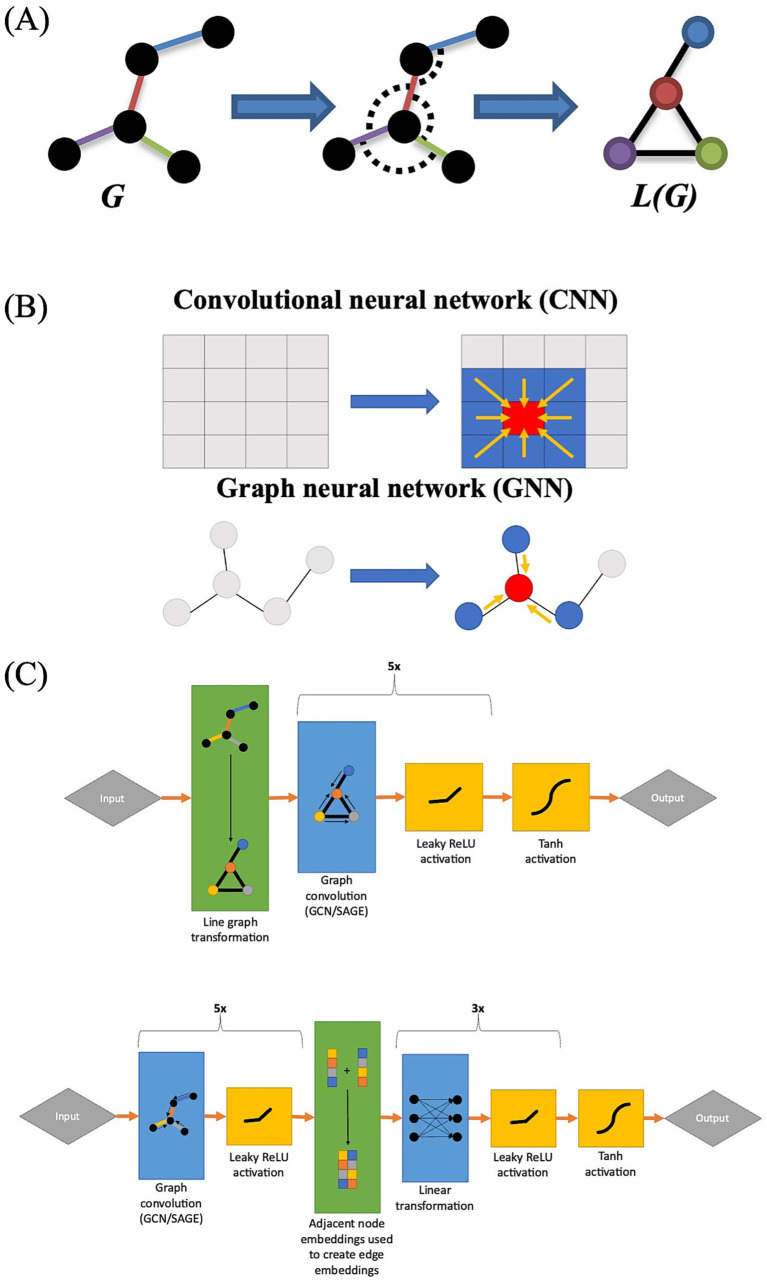
(A) Graphical representation of a line graph transformation. Note how each edge in graph G corresponds to a node in graph L(G). (B) Graphical representation of the difference between GNNs and CNNs, with CNNs integrating information from adjacent cells while GNNs integrate information from adjacent nodes. (C) Architecture of line GNNs (top) and the architecture of traditional GNNs for FC analyses (bottom).

A line graph is a type of derivative graph in which the edges of the original graph are treated as nodes in the line graph (Cai et al., 2022) (Figure 1A). In computational chemistry and biomolecular interaction analysis, the use of line graphs in GNNs has proven effective in edge prediction, as line graphs permit the user to explicitly incorporate relational data into their models (Choudhary and DeCost, 2021; Zheng et al., 2022; Han and Zhang, 2023). In computational chemistry, line graphs allow researchers to represent atomic bond angles as nodes, whereas in biomolecular interaction analysis, they allow researchers to represent individual interactions as nodes (Choudhary and DeCost, 2021; Zheng et al., 2022; Han and Zhang, 2023). Given that FC analyses are edge-based, we also investigated the effectiveness of using line graph-based GNNs to predict changes in FC that occur during an auditory language task. We chose to utilize two common GNN architectures: a graph-based version of convolutional neural networks called graph convolutional networks (GCN) proposed by Kipf and Welling (2017) (Figure 1B) and an extension of this architecture called GraphSAGE (Kipf and Welling, 2017; Hamilton et al., 2017). We hypothesize that GNNs trained on FC line graphs (that is, functional correlations as nodes connected by ROIs) will show improved performance over GNNs trained on traditional FC networks.

## 2 Methods

### 2.1 Data collection

We performed cross-validation on two datasets to assess model performance: dataset #1, which included 205 participants, and dataset #2, which included 12 participants from a different experiment.

#### 2.1.1 Dataset #1

Task-based fMRI data for 205 healthy adult individuals were queried from the Human Connectome Project (HCP) database. The subjects were matched for age, education, and handedness and performed an auditory language comprehension task.

Subjects were asked to perform an experimental task in which they listened to a story and answered questions about the story’s topic. For example, after auditorily listening to a story about an eagle rescuing a man after he had done the same for the eagle, the participant may be asked “Was the story about revenge or reciprocity?” They were asked to register their responses by pressing a button box. Similarly, as a baseline control study, subjects were asked to solve an auditorily presented mathematical problem, such as “Four plus twelve minus two plus nine. Does this equal twenty-two or twenty-three?” The mathematical and language tasks are described in further detail in Binder et al. (2011) and Barch et al. (2013), with the examples adapted from Binder et al. (2011). For this data collection, there were two 3.8 min imaging acquisition sessions (a total of 7.6 min). Each session contained four ~1 min epochs in which story stimuli were presented and four ~1 min epochs of math calculations as described above. The authors reported that in cases in which subjects completed the math calculations with residual time remaining within the epoch, additional math calculations were performed in order to fill the time as needed. They used the mathematical task data as control to negate the neuroactivational contributions from nonlinguistic cognitive resources such as attention and executive memory as well as scanner and physiologic noise.

BOLD neuroactivation data were acquired noninvasively using gradient-echo EPI on a 3 T MRI with a FOV of 208-x 180 mm; slice thickness of 2.00×2.00×2.00 mm voxels and multiband factor of 8. The TR was 720-ms and TE of 33.1 ms and a Flip angle of 52 degrees.

#### 2.1.2 Dataset #2

To assess the model’s generalizability, task-based fMRI data for twelve healthy controls recruited from the NINDS Epilepsy Unit under IRB approved protocol 14-N-0061 were included in this secondary retrospective analyses. The twelve subjects were matched for education, age, and handedness. Further demographic information can be found in Table 1.

**Table 1:** Demographics of study participants with percentages in parentheses.

Characteristic	Dataset #1: *n* (%)	Dataset #2: *n* (%)
Age		
22–25	73 (35.6)	6 (50.0)
26–30	75 (36.6)	1 (8.3)
31+	57 (27.9)	5 (41.7)
Race		
Asian/Hawaiian/Pacific Is.	36 (17.6)	–
Black or African American	42 (20.5)	4 (33.3)
White	111 (54.2)	5 (41.7)
Other/Unknown	16 (8)	3 (25.0)
Ethnicity		
Hispanic/Latino	24 (11.7)	–
Not Hispanic/Latino	179 (87.3)	–
Unknown	2 (1.0)	–
Gender		
Female	106 (51.7)	6 (50.0)
Male	99 (48.3)	6 (50.0)
Handedness		
Left	21 (10.2)	2 (16.7)
Right	182 (88.8)	10 (83.3)
Neither	2 (1.0)	–

These subjects performed a different auditory language comprehension task from the one performed in the HCP dataset. In this task, called an auditory description decision task (ADDT), subjects listened to a descriptive sentence of an item from the Boston Naming Test which was either true (“a large gray animal is an elephant”) or false (“spaghetti is something you sit on”) (Rolinski et al., 2020). Subjects answered yes or no using a button box. The baseline task for the second task was also different from the HCP dataset in that subjects were asked to listen to sentences played in reverse order. By using reversed speech as the baseline control, no syntactic or semantic information is conveyed when the subject listens to the stimuli. The associated neuroactivation in the control task, therefore, only represents listening to sound that is devoid of linguistic content.

Similar to the first dataset, the paradigm for the second dataset also followed a block design. Five epoch cycles where each cycle consists of an alternating block of task and control task was performed. Each epoch was repeated for a 30 s duration followed by the control task for a total scanning time of 2.5 min for the experimental task and 2.5 min for the control task.

Imaging acquisition was performed on a 3 T MRI scanner at the National Institutes of Health NMR Center using a 32-channel head coil. Imaging parameters were flip angle: 65 degrees; TR: 2000 ms, TE: 30; Voxel size: 3 mm x 3 mm x 4 mm; FOV: 216 × 216 mm; slice thickness of 4 mm. During resting state fMRI, participants lay still in the scanner while staring at a fixation mark in the display for the duration of the scan. During task based fMRI, participants were asked to perform an ADDT in which they listened to an auditorily presented sentence and then decided whether the following word matched the description. The detail of the tb-fMRI procedure has been described elsewhere (Rolinski et al., 2020; Gaillard et al., 2007).

### 2.2 MRI preprocessing

MRI preprocessing was performed per previously reported techniques (RaviPrakash et al., 2021). Specifically, we preprocessed the anatomical data to achieve accurate surface segmentation. We performed surface parcellation using T1 weighted MPRAGE and fluid-attenuated inversion recovery (FLAIR) images using FreeSurfer (RaviPrakash et al., 2021; Fischl, 2012). We registered the MPRAGE volume with the MNI-305 atlas using an AFNI registration to perform the cortical surface parcellation. We then performed skull stripping. We then followed the intensity gradients between the white- and gray-matter to generate surface segmentations for each hemisphere. Similarly, we generated the pial surface using the intensity gradients between the gray matter and cerebral spinal fluid. We used the different contrast in the FLAIR images to further define the pial surface segmentation, after which surface labeling was done as in Desikan et al. (2006).

### 2.3 fMRI preprocessing

We used Analysis of Functional NeuroImages toolbox (AFNI) to preprocess the fMRI data and used surface-based cortical analysis pipelines (Cox, 1996; Cox and Hyde, 1997). Pre-steady state volumes prior to reaching equilibrium magnetization were discarded. First, we conducted slice timing correction to synchronize timing across brain slices and then performed motion correction by setting the motion threshold to 0.3 mm. We censored BOLD signal that exceeded this threshold. We applied a regress bandpass filter tb-fMRI with a frequency of 0.01–0.10 Hz, to further surpass signal noise and then applied a 6 mm spatial smoothing kernel to further reduce the noise. We then added up the coherent signals as previously described (RaviPrakash et al., 2021).

The rs-fMRI preprocessing was nearly identical to the tb-fMRI preprocessing, including the use of a 6 mm spatial smoothing kernel. We used regress bandpass filter for rs-fMRI, with a frequency range of 0.01–0.5 Hz, to further eliminate noise.

### 2.4 Functional connectivity network analysis

The task network is generated by first concatenating the time-series of all task trails/blocks in a single subject. We then computed Spearman correlation to generate the static FC. We included all statistically significant task correlations compared to baseline, providing that the functional correlations were present in at least a certain percentage of subjects. We empirically calculated the participation threshold to be 85%. The associated *p*-values are corrected using Bonferroni correction. Of note, many investigators apply an edge strength threshold to ensure the inclusion of robust functional correlations in the statistical analyses. However, this can inadvertently eliminate functionally relevant functional correlations from our analyses. In order to minimize this occurrence, we included all significant (*p* < 0.05 post-Bonferroni correction) connections across sample dataset. But then applied a participation threshold to ensure that the correlations were robust and reproducible. This enables us to avoid arbitrarily cutting off the functional correlations with correlation strengths that may be relevant for a particular individual’s FC performance.

This left us with 32 ROIs connected by 71 functional correlations for the first dataset and 20 ROIs connected by 12 functional correlations for the second. The static FC graphs generated were then implemented as NetworkX objects in Python (Hagberg et al., 2008).

#### 2.4.1 Model creation

Four machine learning architectures were assessed for their predictive capabilities. The first two were graph convolutional architectures implemented using a traditional GCN as well as the GraphSAGE architecture in which the nodes were embedded with per-timepoint ROI activity levels at baseline (Figure 1C). In the traditional GCN, the edges were embedded with functional correlation measurements at baseline (Figure 1C). These models use the traditional representation of FC networks as ROIs (nodes) connected by functional correlations (edges).

The final two compared GCN and GraphSAGE architectures that used a line graph configuration. In line graphs nodes (ROI) are converted to edges and FC network edges are represented as nodes. The line GCN included per-timepoint ROI activation data embedded in its edges, whereas the line GraphSAGE discarded this information because the architecture did not permit edge attributes to be incorporated. All neural networks were implemented using the PyTorch Geometric package (Fey and Lenssen, 2019).

All models were trained using the mean squared error (MSE) loss function. All neural networks were trained using the Adam optimizer over the course of 100 epochs, or training cycles. Apart from the line graph GCN, which had a learning rate of 1×10-3, all neural networks had a learning rate of 2×10-6. A higher learning rate was chosen for the line graph GCN because it was found to train far more slowly than the other models otherwise.

Each model had either five GCN or five GraphSAGE convolutional layers depending on model type with 512 neurons per layer, and there was a negative slope Leaky ReLU activation function of 0.05 between each layer. The models without line graph transformations also included a two-layer linear perceptron at the end to convert node embeddings to edge embeddings. A Tanh activation function was applied at the end of each model to transform the output to a (−1.0, 1.0) range.

The performance of each model was assessed using five-fold cross-validation. Benchmark error was calculated by creating a set of predicted values for a set of participants in the training set and applying this prediction to the validation set. We decided to use this metric to determine whether the models were creating truly personalized predictions, or they were simply converging on an average value for each functional correlation. The accuracy was quantified using MSE.

#### 2.4.2 Statistical methods

Statistical testing was performed using SciPy version 1.9.0, and we determined the significance level to be *p* < 0.05. Comparisons between benchmark and validation performance for each model, as well as comparisons in performance between models, were performed through a Wilcoxon signed rank test. Comparisons in the time taken to train each model were performed using a paired t-test. Both analyses that involved comparing each model to each other model were corrected for multiple comparisons through Bonferroni correction.

## 3 Results

**Figure 2:**
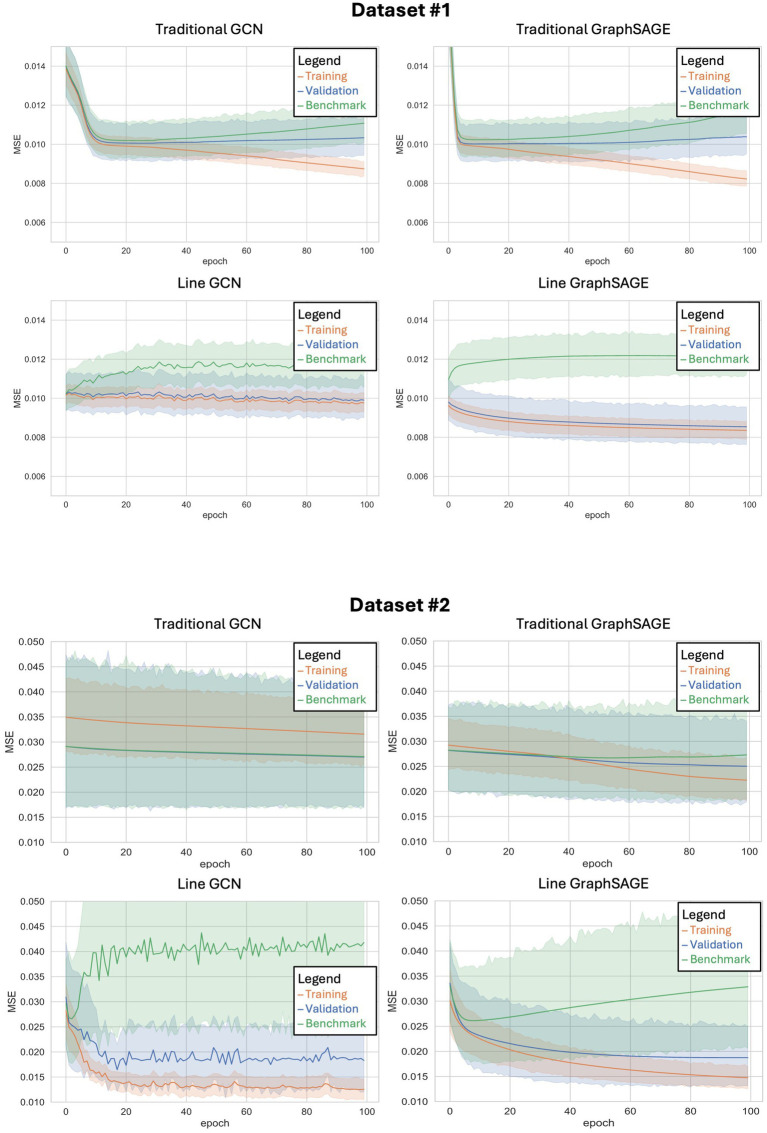
Training curves for each of the four neural networks tested when applied to dataset #1 (top) and dataset #2 (bottom). The x-axis shows the number of epochs (or training cycles) that have occurred, while the y-axis shows the mean squared error for each epoch. The shaded region represents the 95% confidence interval.

All four models were found to outperform their benchmarks with the HCP dataset and the second dataset, indicating that all of them were making individualized predictions (Figure 2; Table 2). After comparing each model’s validation performance to its benchmark performance, we examined their performance relative to one another. In the HCP dataset, we found that the line graph-based GCN, traditional GCN, and traditional GraphSAGE models did not show any statistically significant differences from each other; however, we did find that the line GraphSAGE showed a significant improvement – defined as a decrease in MSE – over all other tested architectures (*p* < 0.0001 in all cases).

**Table 2:** Performance of each machine learning architecture tested.

		Traditional GCN	Traditional GraphSAGE	Line GCN	Line GraphSAGE
Dataset #1	Benchmark MSE	0.0111	0.0116	0.0115	0.0122
	Validation MSE	0.0103	0.0104	0.0099	**0.0085**
	Difference	−0.0007	−0.0013	−0.0016	**−0.0036**
	*P*-value	*0.0011*	*0.0005*	*<0.0001*	*<0.0001*
	Training time (s)	**310.7**	382	500.5	479.5
Dataset #2	Benchmark MSE	0.0271	0.0273	0.0418	0.0329
	Validation MSE	0.027	0.025	**0.0183**	0.0187
	Difference	−0.0001	−0.0023	**−0.0235**	−0.0142
	*P*-value	0.2324	0.1391	*0.0005*	*0.0068*
	Training time (s)	20.1	26.3	**17.1**	20.8

One-way *t*-test used to determine significance. Significant *p*-values (<0.05) are listed in italics, while superior results for training time, validation MSE, and benchmark-validation MSE are listed in bold.

When applied to the second dataset, using a different set of auditory language tasks, similar patterns emerged. The line GCN and the line GraphSAGE models demonstrated significantly improved performance over their traditional counterparts, with notable reductions in MSE and significant *p*-values (line GCN: *p* = 0.0005; line GraphSAGE: *p* = 0.0068), underscoring their robustness across varied datasets (Table 2; Figure 2). We performed paired *t*-tests comparing the validation MSEs across each fold. The *p*-value between traditional GCN and line GCN was 0.004953, indicating a statistically significant difference in their performance, with the line GCN model consistently outperforming the traditional GCN. Similarly, the paired t-test comparing traditional GraphSAGE and line GraphSAGE models yielded a *p*-value <0.003.

With the HCP dataset, both line graph-based neural networks immediately show a divergence between validation and benchmark performance early on in their training, and this difference in MSE only grows as more training epochs are completed (Figure 2). Additionally, these models show a striking resistance to overfitting, as their training error remains only marginally lower than their validation error even as the number of epochs increases. This is in stark contrast to the models without line graph transformations, both of which show an increase in training accuracy coupled with a decrease in validation accuracy in their later epochs – a widely recognized hallmark of overfitting.

As observed with the HCP dataset, both line graph-based neural networks initially diverged from the benchmark performance when trained on the second dataset (Figure 2). However, the degree of divergence in MSE between the training and validation phases was less pronounced. The line graph-based networks maintained a certain degree of resilience against overfitting, with the line GCN and line GraphSAGE models consistently demonstrating smaller gaps between training and validation errors across epochs. This adaptability was less evident in the traditional models, which, despite improvements, exhibited a greater tendency towards overfitting in the second dataset compared to the HCP dataset.

Training times varied significantly across models, with line GNNs requiring more time compared to traditional models for the HCP dataset. We found that line GNNs took significantly more time to train than traditional GNNs (line GraphSAGE and line GCN vs. traditional GCN: *p* < 0.0001; line GraphSAGE vs. traditional GraphSAGE: *p* = 0.0004; line GCN vs. traditional GraphSAGE: *p* = 0.0002). The traditional GraphSAGE trained significantly slower than the traditional GCN (*p* = 0.0001); however, the line GraphSAGE and line GCN did not show a statistically significant difference in training time from each other (*p* = 0.5150) (Table 2).

The training times for the second dataset revealed additional insights. Line GCN and line GraphSAGE models did not exhibit significant differences in training duration compared to the traditional models (Table 2). The traditional GraphSAGE exhibited a slower training time compared to the traditional GCN; however, the contrast in training times between the line and traditional models was not as pronounced with the second dataset as with the HCP dataset.

## 4 Discussion

Specific language deficits in patients with acquired CNS injuries have been well documented in the literature, with the first reported cases dating back to the 19th century. Speech and language deficits have been reported in patients with lesions in Broca’s and Wernicke’s areas, for example. Aphasias have also been reported in patients with injuries that disrupt the pathways between designated ROIs such as in conduction aphasia or global aphasia. However, understanding the mechanisms by which these language deficits arise is essential in developing a robust mechanistic understanding of large scale integration of brain’s highly integrated structural but also functional networks. Understanding how the effects of local injury may permeate throughout the brain that can lead to novel approaches in neurorehabilitation.

The development of neurocomputational models of the neural basis of language processing in the healthy human brain may be an important step in eventually understanding these mechanisms. GNNs are a type of network architecture that serve as a natural deep learning tool by which to characterize the brain’s static functional connectivity changes associated with cognitive behavior. Although it is beyond the scope of this paper to evaluate the performance of all graph-based neural networks, we specifically compared the performance of four graph based models; namely two traditional graph based architectures versus two line graph network architectures using two specific cognitive tasks with two different baseline datasets as input. We were particularly interested in assessing line graph architectures because they enable one to emphasize the “connections” between ROIs, rather than on the ROIs themselves as is typical of traditional graph base models.

In both the HCP dataset and the second dataset, we found that line GNNs outperform traditional GNNs in predicting changes in FC when using baseline FC as its input. Of specific interest, both datasets used fundamentally different baseline data as its input. The HCP dataset used FC associated with word based mathematical decisions. The model successfully predicted the static FC changes associated with language related computations. It demonstrated gradual decreased error rates over time with little to no overfitting patterns when compared to traditional GNNs.

Even when the model architectures were trained on a second dataset using a nonlinguistic baseline, both line GCN and line GraphSAGE models showed statistically significant improvements in MSE compared to the traditional models, suggesting a improved model performance across diverse datasets. However, it is important to note that all models registered higher MSEs when applied to the second dataset, a testament to the influence of dataset size and heterogeneity on the model’s performance. That said, one of the important elements of the model’s performance was its consistency in modeling the FC changes with no overfitting.

More recent studies have applied GNN machine learning models to characterize ROI activation in fMRI where ROIs are indicated as nodes and the connections linking the nodes as edges. We compared traditional GCN models to a similar common architecture of GNNs, called GraphSAGE. In the case of traditionally constructed FC data, we found that the GraphSAGE model performed similarly to the GCN model. However, we found that both models trained on traditional FC suffered from the problem of overfitting; this was particularly true for the GraphSAGE model.

When making predictions, GNNs tend to weight node-based data particularly heavily, with some common architectures (such as GraphSAGE) even omitting edge attributes altogether. This makes feature selection challenging when the most relevant features are edge-based, as is the case with FC studies, which in turn prompted our investigation of line graph-based methods. Indeed, we found that line graph models – namely the line GraphSAGE model – resulted in improved modeling of our data compared to the traditional GNN models, regardless of the context of the specific language-based task. The line graph models for both types of very different data inputs resulted in accurate modeling of the FC changes for two very different types of language-related changes with little to no data overfitting. This supports our hypothesis that line graph models would better characterize connections between ROIs, possibly because of inherent feature selection, which is a common method to avoid overfitting (Lever et al., 2016). Because the line graph construction means that line GNNs discard much of the ROI-based activation and structural information in the original graph, and even omit this information entirely in the case of the line GraphSAGE model, the information that does remain is FC data which is more likely to be relevant to the target predictions. Thus, overfitting on less-relevant ROI data is mitigated with line graph models, which could explain the line GNNs’ improved performance with respect to overfitting.

Although our findings are encouraging vis a vis the potential for using line graph-based models to accurately model changes in cognitive states, several caveats warrant mentioning: (1) We only used static FC as our input source to generate the model. (2) Like most deep neural networks, line graph-based models are still “black box” architectures and for these reasons future research will be needed to future develop new research tools, such as the addition of GNN explainers to make line graph based modeling more interpretable and explainable (Yuan et al., 2020) because explainable AI models will be essential in understanding structural perturbations that result from CNS injuries. (3) We found that line GNNs had longer training times than traditional GNNs when trained on the HCP dataset, which could imply a lack of scalability.

In conclusion, we found that line graph-based models can serve as powerful computational tools to model changes in static network correlations associated with different mental states as assessed here using two different tasks and to be able to do so with little to no overfitting, an important characteristic. That said, we think that the typology of graph-based in general can be exploited to better understand the dysfunctional relationships between local clusters of locally vs. long-distance affected functional correlations in whole brain network connectivity datasets, especially as affected in individual patients and patient populations. Further research comparing the performance of line GraphSAGE modeling to other graph-based models such as Bayesian networks is still warranted followed by the use of computer simulations to assessing the model’s performance in predicting network connectivity changes following CNS injury. Importantly, these models suggest that novel computational approaches that not only classify patients but provide the basis for improved mechanistic understandings of local and long-ranging network correlations changes following CNS injury may be possible. This provides the basis for a novel focus of research that uses these simulations to develop promising novel mechanistic therapeutic interventions in the future.

## Data Availability

The data analyzed in this study is subject to the following licenses/restrictions: the original raw imaging datasets are from the Human Connectome Project and are accessible publicly with the appropriate permissions granted from Washington University. The code itself is available through NIH/BTRIS data repository and can be shared with the approved request. In addition, the anonymized fMRI data for the second dataset is also available permission from NIH through the NIH/BTRIS data repository. Requests to access these datasets should be directed to Nadia Biassou, biassoun@nih.gov.
